# Study on the Improvement Effect of Polypropylene Fiber on the Mechanical Properties and Freeze–Thaw Degradation Performance of High Fly Ash Content Alkali-Activated Fly Ash Slag Concrete

**DOI:** 10.3390/polym17020175

**Published:** 2025-01-13

**Authors:** Zhu Yuan, Yanmin Jia, Junming Xu

**Affiliations:** School of Civil Engineering and Transportation, Northeast Forestry University, Harbin 150040, China; zhu_yuan163@163.com (Z.Y.); xjm_2021@126.com (J.X.)

**Keywords:** alkali-activated fly ash slag concrete, polypropylene fiber, high fly ash content, room temperature preparation, freeze–thaw degradation

## Abstract

This article systematically investigated the improvement effect of polypropylene fiber (PPF) on the mechanical and freeze–thaw properties of alkali-activated fly ash slag concrete (AAFSC) with high fly ash content and cured at room temperature. Fly ash and slag were used as precursors, with fly ash accounting for 80% of the total mass. A mixed solution of sodium hydroxide and sodium silicate was used as alkali activator, and short-cut PPF was added to improve the performance of AAFSC. Firstly, the strength characteristics of AAFSC at different curing ages were studied. Then, key indicators such as morphology, residual compressive strength, weight loss, relative dynamic modulus of elasticity (RDME), and pore characteristics of AAFSC after different freeze–thaw cycles were tested and analyzed. The strength performance analysis showed that the optimal dosage of PPF was 0.90%. When the alkali equivalent of the alkali activator was increased from 4% to 6%, the frost resistance of AAFSC could be improved. Furthermore, adding 0.90% PPF could increase the freeze–thaw cycle number of AAFSC by about 50 times (measured by RDME). With the increase in freeze–thaw cycles, the porosity of AAFSC increased, the fractal dimension decreased, and the proportion of harmless and less harmful pores decreased, while the proportion of harmful and multiple harmful pores increased. The relationship model between the porosity and compressive strength of AAFSC after freeze–thaw cycles was established.

## 1. Introduction

With the continuous construction of transportation infrastructure such as highways and bridges in China, the demand for cement concrete, as one of the main building materials, continues to increase, leading to a sustained increase in cement production. In 2023, the national cement production reached 2.023 billion tons. Such a huge cement production comes with significant environmental costs. According to statistical data, for every ton of cement produced, approximately 0.8 tons of CO_2_ are emitted into the atmosphere [1,2]. The annual CO_2_ emissions from the cement manufacturing industry account for about 5–8% of global CO_2_ emissions, and industrial energy consumption accounts for about 10–15% of global energy consumption [3,4,5]. It is crucial to develop new cementitious materials to replace cement and reduce its carbon footprint.

With the acceleration of China’s industrial process, the discharge of industrial solid waste has been increasing year by year. According to statistical data, in 2023, China’s fly ash production reached 899 million tons, accounting for approximately 55% of the total global production, and the total production of granulated blast furnace slag was over 60 million tons. The storage of such a large amount of bulk industrial solid waste not only damages the ecological environment, but also occupies a large amount of natural resources such as farmland, forest land, and grassland. At present, the productization and utilization of typical bulk industrial solid waste are subject to multiple challenges such as technological constraints, lack of standards for recycled products, and limited application markets and transportation radii. Compared with the huge production and historical storage volume, the amount of industrial product utilization is relatively limited. Relying on the production of these bulk industrial solid wastes into industrial products is far from enough. Currently, it is urgent to explore new ways for the large-scale comprehensive utilization of typical bulk industrial solid wastes such as fly ash and slag.

Fly ash and granulated blast furnace slag contain abundant volcanic ash components such as calcium oxide, silicon dioxide, and aluminum oxide. Fly ash also contains a large amount of amorphous silicon aluminum substances [6], so it can be used as a precursor to prepare alkali-activated materials.

From previous studies, slag-based alkali-activated materials have poor workability, short setting time, and large shrinkage [7,8,9,10]. Due to the high excitation energy of fly ash, pure fly ash-based alkali-activated cementitious materials usually require high-temperature curing [11,12,13,14,15,16], microwave curing [17,18,19,20,21,22], or direct current curing [23,24,25,26] to accelerate the reaction process of the alkali-activated fly ash system and obtain satisfactory mechanical properties. However, these curing methods consume more energy, increase construction costs and technical difficulties, and are difficult to apply in practical engineering, which may reduce their applicability in the field of civil engineering. Therefore, achieving room temperature curing of alkali-activated materials is crucial for their large-scale promotion and application in practical engineering [27,28].

By utilizing the synergistic effect of fly ash and slag in alkali-activated reactions [29,30], alkali-activated fly ash slag cementitious materials prepared at room temperature can achieve good strength and other properties [31,32,33,34,35]. Some scholars have attempted to improve the strength and other properties of alkali-activated concrete by incorporating short-cut fibers [36,37,38,39,40]. Although AAFSC has shown broad application prospects, there is still a lack of systematic comprehensive research on its mechanical and durability properties.

Frost resistance is one of the key indicators of concrete durability. At present, there is a lack of research on the freeze–thaw degradation and damage mechanism of AAFSC, which seriously hinders the application of this green building material in cold regions.

This study focused on the improvement of the mechanical properties and freeze–thaw durability of AAFSC by PPF. The research results will provide an important scientific basis for the application of AAFSC in the construction of transportation infrastructure in cold regions. Not only can it help reduce the use of cement in engineering construction and achieve efficient utilization of industrial solid waste, but it also has profound significance for promoting the sustainable development of the concrete industry.

## 2. Raw Materials and Mix Proportions

### 2.1. Raw Materials

The precursors were first-grade fly ash and S95-grade slag; their chemical compositions are shown in Table 1. The alkaline activator was a mixed solution of sodium silicate and sodium hydroxide. In liquid sodium silicate, the content of silicon dioxide was 32.35% (percentage by weight), the content of sodium oxide was 13.73% (percentage by weight), the density was 1.53 g/mL, and the water content was 53.92%. Sodium hydroxide was a white opaque solid with a purity of not less than 96%. The fine aggregate was medium sand with a fineness modulus of 2.66, and the coarse aggregate was 5–20 mm continuously graded crushed stone. The length of PPF used was 12 mm; the appearance and main parameters of PPF refer to the author’s previous research [41]. The water reducer used was a naphthalene-based water reducer.

### 2.2. Mix Proportions

In the mix proportions of AAFSC, the proportion of fly ash to the total mass of the precursor was 80%. The modulus of the alkaline activator was 1.0, and three different alkaline equivalents were set: 4%, 6%, and 8%. The PPF was added to concrete with an alkali equivalent of 6%, with fiber content of 0.45%, 0.90%, and 1.35% (volume fraction), respectively. The mix proportions of AAFSC are shown in Table 2. In the “MIX ID”, for example “A6M1PPF0.45”, where “A6” represents an alkali equivalent of 6%, “M1” represents an activator modulus of 1.0, and “PPF0.45” represents a PPF content of 0.45% by volume fraction.

## 3. Specimen Preparation and Curing

**Figure 1:**
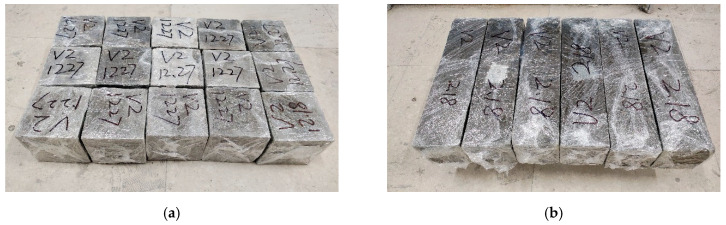
Concrete specimens cured at room temperature. (**a**) Cube specimens with dimensions of 100 mm × 100 mm × 100 mm. (**b**) Prism specimens with dimensions of 100 mm × 100 mm × 400 mm.

The fresh concrete was mixed evenly, it was compacted with a vibration table after molding, and the surface was covered with plastic wrap to prevent moisture evaporation. After 24 h of being placed in the mold, the specimens were unmolded and wrapped tightly with plastic film (as shown in Figure 1). At room temperature (20 ± 2 °C) in the laboratory, the concrete specimens were cured to the specified age.

## 4. Test Procedures

### 4.1. Measurement of Strength

According to the standard GB/T 50081-2019 [43], the compressive strength and flexural strength of the concrete specimens were tested, and the strength values were calculated.

### 4.2. Freeze–Thaw Cycles Test

Taking into account the compressive strength and flexural strength of AAFSC without fiber addition, as well as the improvement effect of fiber addition on the strength characteristics of AAFSC, A4M1, A6M1, and A6M1PPF0.90, sets of concrete specimens were selected for rapid freeze–thaw testing. The test specimens consisted of cubes with dimensions of 100 mm × 100 mm × 100 mm and prisms with dimensions of 100 mm × 100 mm × 400 mm. Cube specimens were used to test the compressive strength, and prism specimens were used to test the changes in weight, RDME, and pore characteristics after freeze–thaw cycles.

**Figure 2:**
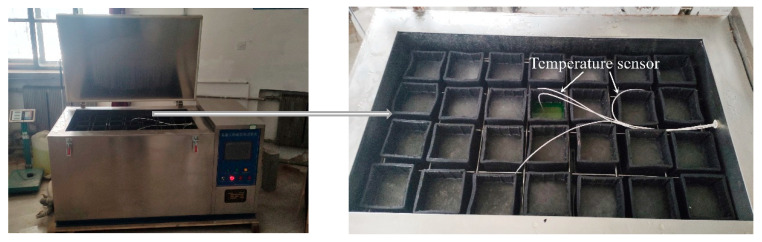
Rapid freeze–thaw test equipment for concrete.

The TDRF-I type concrete rapid freeze–thaw test equipment (as shown in Figure 2) produced by Tianjin Huida Experimental Instrument Factory was used for the freeze–thaw cycles test of the concrete. According to the standard GB/T 50082-2009 [44], during the test, the highest temperature at which the center of the test specimens melted was (5 ± 2) °C, and the lowest temperature at which they froze was (−18 ± 2) °C. A freeze–thaw cycle was completed approximately every 4 h. The experiment was stopped when one of the following situations occurred: (1) the number of freeze–thaw cycles reached 300; (2) the RDME of the specimens decreased to 60%; (3) the weight loss of the specimens reached 5%.

### 4.3. Residual Compressive Strength Test

The compressive strength of cubic specimens subjected to different freeze–thaw cycles was tested using the same testing equipment and parameter settings as described in Section 4.1.

### 4.4. Weight Loss Test

The weight of the prismatic specimens subjected to different freeze–thaw cycles was tested using an electronic scale.

### 4.5. RDME Test

**Figure 3:**
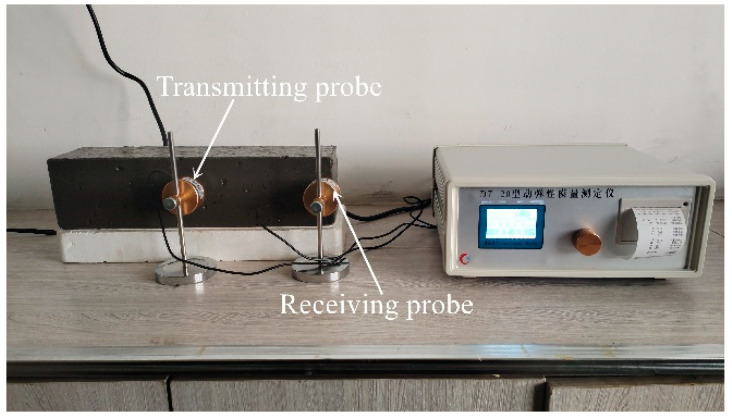
RDME tester for concrete.

The RDME of the prismatic specimens subjected to different freeze–thaw cycles was tested using the DT-20 dynamic modulus tester (Zhongbo Ruike, Cangzhou, Hebei, China) (Figure 3).

### 4.6. Pore Characteristic Test

The pore characteristics of concrete specimens with different freeze–thaw cycles were tested using mercury intrusion porosimetry (MIP). The testing instrument used was the McAutopore V9620 (Micromeritics Norcross GA US) mercury intrusion porosimetry tester with a contact angle of 130°.

## 5. Results and Discussion

### 5.1. Compressive Strength

**Figure 4:**
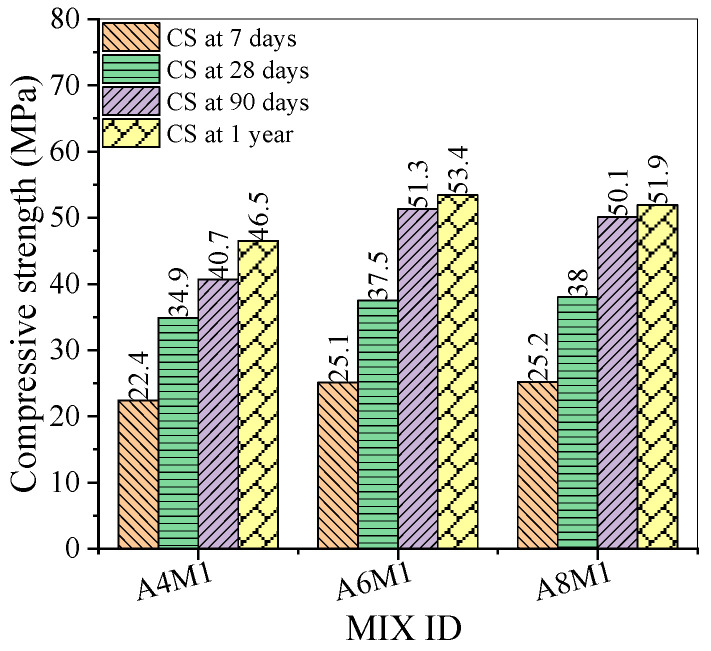
Compressive strength of AAFSC without fiber addition.

Figure 4 shows the compressive strength of AAFSC without fiber addition at different curing ages. As the alkali equivalent increased, the compressive strength of concrete at 7 and 28 days increased, while the compressive strength at 90 days and 1 year first increased and then decreased. Notably, the compressive strength of A6M1 group concrete at 7 and 28 days was 25.1 MPa and 37.5 MPa, respectively; at the curing age of 1 year, the compressive strength reached 53.4 MPa, an increase of 42.4% compared to the 28-day strength value.

Under the same dosage of cementitious materials and aggregates, the 7-day and 28-day compressive strengths of ordinary cement concrete were 28.6 MPa and 28.8 MPa, respectively [41]. By comparison, it can be seen that the 7-day compressive strength of AAFSC was lower than that of ordinary cement concrete. This is because the amount of fly ash in the alkali-activated cementitious material used in this study was relatively high, and the reaction process in the first 7 days was slower under normal temperature curing conditions. As the curing time prolonged, the alkali-activated cementitious material continued to react, and by the age of 28 days, the compressive strength of AAFSC exceeded that of ordinary cement concrete.

**Figure 5:**
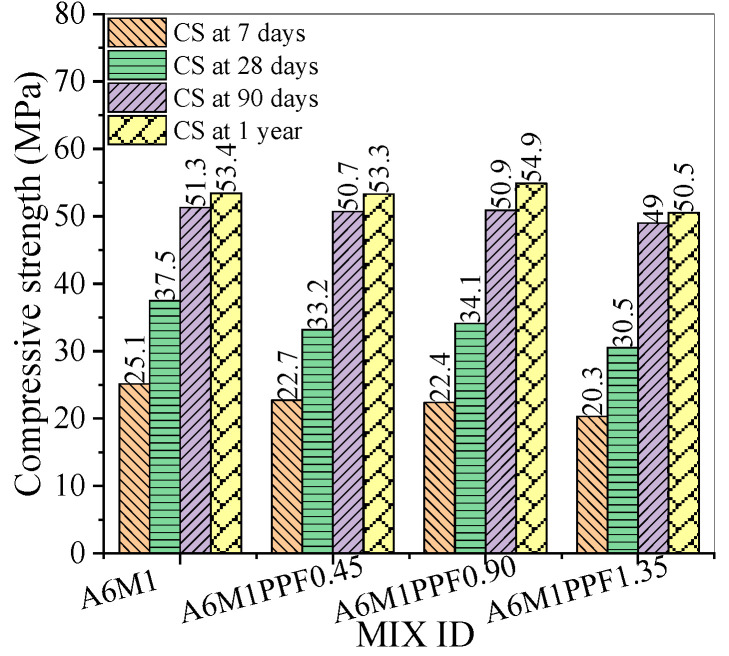
Compressive strength of AAFSC mixed with PPF.

Figure 5 shows the compressive strength of AAFSC mixed with PPF. The compressive strength of the concrete mixed with PPF at 7, 28, and 90 days was lower than that of A6M1. This indicated that during the 90-day curing period, the PPF did not effectively improve the compressive strength of AAFSC. When the 1-year curing period was reached, the compressive strength of A6M1PPF0.90 reached 54.9 MPa, which was 2.81% higher than that of A6M1.

### 5.2. Flexural Strength

**Figure 6:**
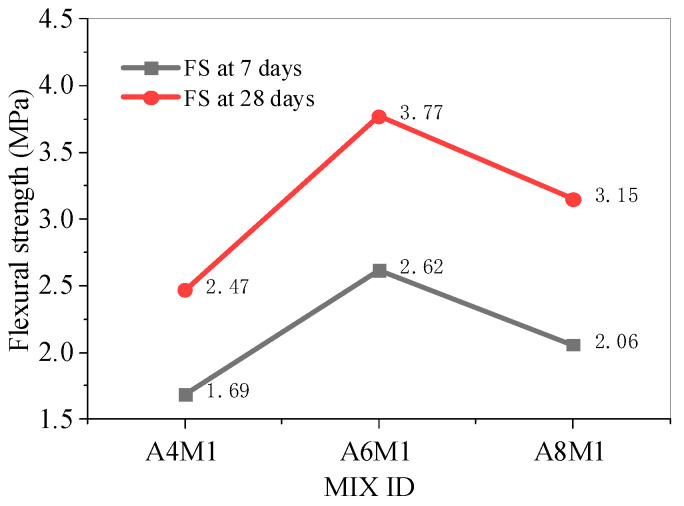
Flexural strength of AAFSC without fiber addition.

Figure 6 shows the flexural strength of AAFSC without fiber addition. As the alkali equivalent increased, the flexural strength first increased and then decreased. Notably, the A6M1 concrete had the optimal flexural strength, with strength values of 2.62 MPa and 3.77 MPa at 7 and 28 days, respectively.

**Figure 7:**
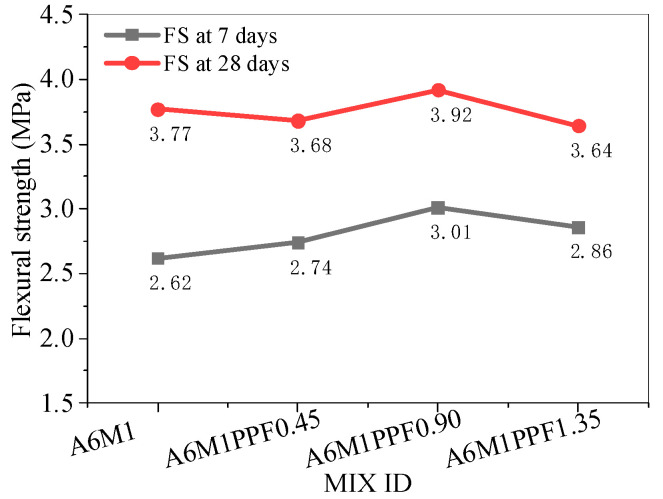
Flexural strength of AAFSC mixed with PPF.

Figure 7 shows the flexural strength of AAFSC mixed with PPF. When PPF was added, the flexural strength first increased and then decreased, with 0.90% being the optimal dosage of PPF. The 28-day flexural strength of A6M1PPF0.90 was 3.92 MPa, which was 3.98% higher than A6M1.

### 5.3. Analysis of Freeze–Thaw Degradation Morphology

**Figure 8:**
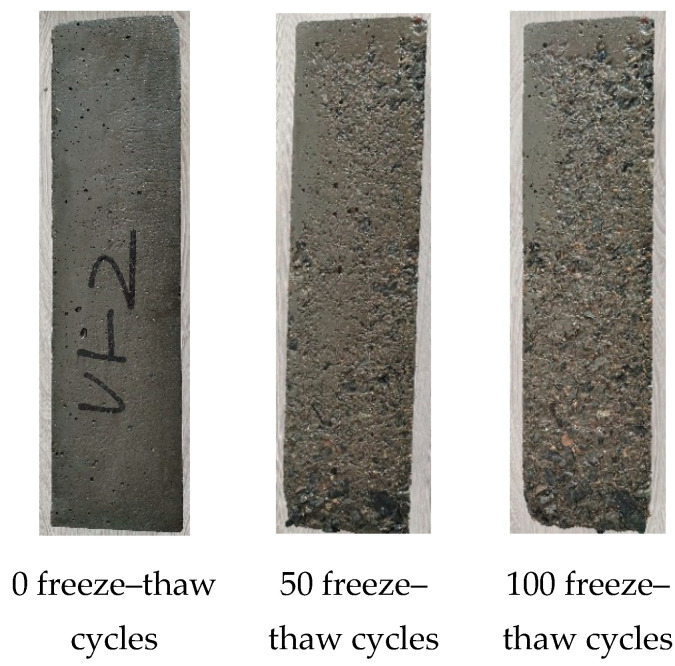
Morphology of freeze–thaw failure of A4M1.

**Figure 9:**
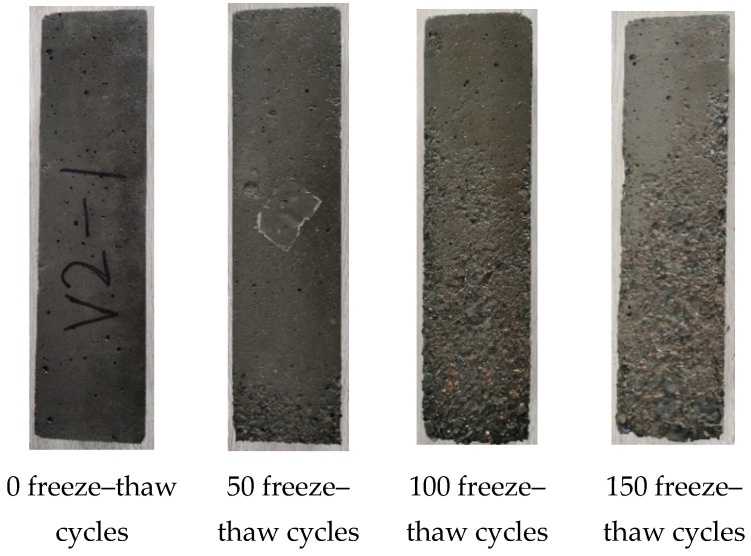
Morphology of freeze–thaw failure of A6M1.

**Figure 10:**
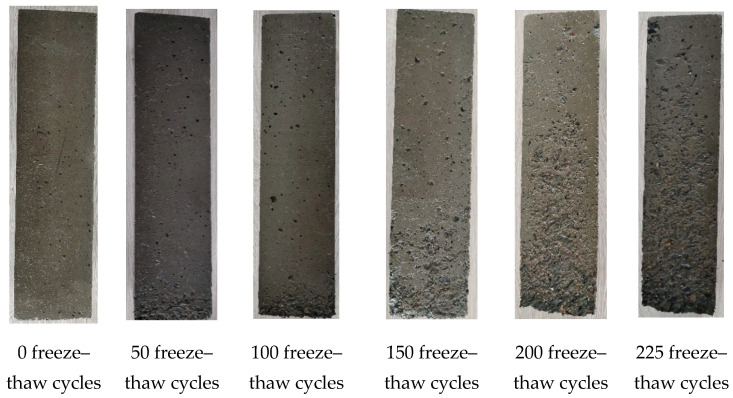
Morphology of freeze–thaw failure of A6M1PPF0.90.

Figure 8, Figure 9 and Figure 10 show the surface failure modes of concrete in groups A4M1, A6M1, and A6M1PPF0.90 after undergoing different freeze–thaw cycles. When not subjected to freeze–thaw cycles, the surface of the specimens was smooth, and only a small number of pores left due to air leakage during the formation of the specimens could be seen. After 50 freeze–thaw cycles, the A4M1 concrete showed peeling of the cementitious matrix on the surface of the specimen, and the closer it was to the bottom of the specimen, the more obvious the peeling of the cementitious matrix. After 100 freeze–thaw cycles, a large area of cementitious matrix peeling occurred on the surface of the specimen, and coarse aggregate was exposed at the corner of the bottom of the A4M1.

After 50 freeze–thaw cycles, A6M1 showed only a small amount of cementitious matrix peeling off at the bottom of the specimen compared to A4M1. After 100 freeze–thaw cycles, the surface degradation morphology of A6M1 was similar to that of A4M1 after 50 freeze–thaw cycles. After 150 freeze–thaw cycles, a small amount of coarse aggregate was clearly exposed at the bottom of A6M1.

After 50 and 100 freeze–thaw cycles, only a very small amount of cementitious matrix at the bottom of the specimens peeled off in A6M1PPF0.90. As the number of freeze–thaw cycles increased, the peeling phenomenon on the surface of the specimens gradually spread to the upper part of the specimens. After 200 freeze–thaw cycles, the peeling phenomenon of the cementitious matrix on the upper part of the specimens was still not obvious. At the corner of the specimen’s bottom, due to insufficient cementitious matrix bonding with coarse aggregate, a small amount of coarse aggregate fell off. After 225 freeze–thaw cycles, there was no significant change in the surface morphology of A6M1PPF0.90 compared to 200 freeze–thaw cycles.

The key to improving the frost resistance of concrete is to prevent water infiltration [36]. Comparing A6M1 and A6M1PPF0.90, it can be seen that adding 0.90% PPF significantly delayed the surface degradation of AAFSC after freeze–thaw, thereby improving its frost resistance.

### 5.4. Compressive Strength of Concrete After Freeze–Thaw Cycles

**Figure 11:**
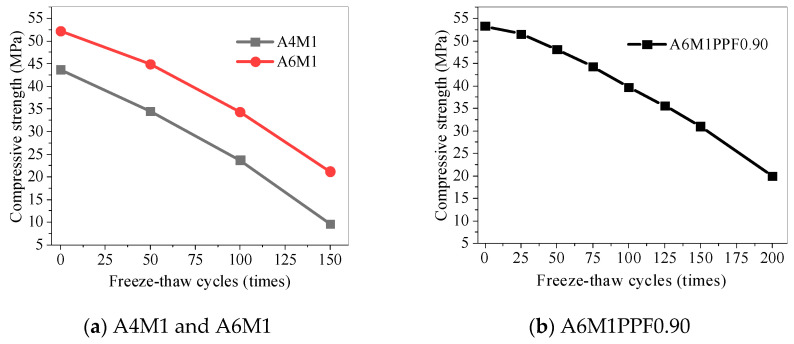
Compressive strength of AAFSC after freeze–thaw cycles.

Figure 11 shows the compressive strength of AAFSC after different freeze–thaw cycles. The compressive strength of A4M1, A6M1, and A6M1PPF0.90 significantly decreased with increasing freeze–thaw cycles. The residual compressive strength of A4M1 and A6M1 after 150 freeze–thaw cycles was 9.6 MPa and 21.2 MPa, respectively, which was equivalent to 22% and 40.6% of the strength at 0 freeze–thaw cycles. The compressive strength of A6M1PPF0.90 could maintain 57.9% of its initial value after 150 freeze–thaw cycles, and there was still 38.3% residual strength after 200 freeze–thaw cycles, indicating that the addition of PPF effectively improved the compressive strength degradation performance of AAFSC after freeze–thaw cycles.

### 5.5. Weight Loss of Concrete After Freeze–Thaw Cycles

**Figure 12:**
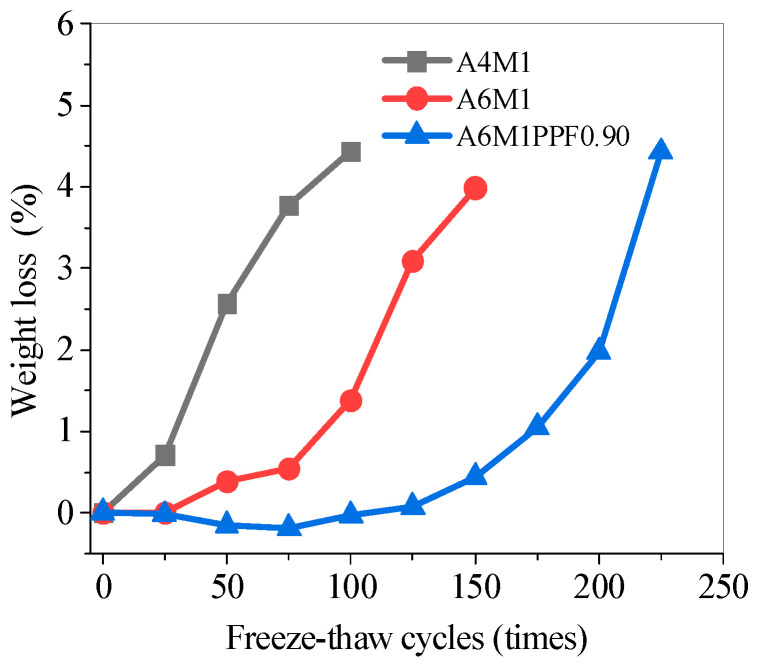
Weight loss of AAFSC during freeze–thaw cycles.

Figure 12 shows the weight loss test results of the AAFSC after freeze–thaw cycles. The weight loss of A4M1 and A6M1 increased with the increasing freeze–thaw cycles. A4M1 had the fastest weight loss rate, reaching 4.43% after 100 freeze–thaw cycles, and exceeding 5% after more than 100 freeze–thaw cycles. The weight loss of A6M1 during the first 100 freeze–thaw cycles was significantly smaller than that of A4M1, with a weight loss of 0.54% after 75 freeze–thaw cycles and 1.38% after 100 freeze–thaw cycles. After 100 freeze–thaw cycles, the weight loss of A6M1 significantly increased, reaching 3.99% after 150 freeze–thaw cycles and exceeding 5% after 175 freeze–thaw cycles.

During the initial stage of freeze–thaw cycles, the A6M1PPF0.90 showed a negative weight loss, indicating an increase in weight. After 75 freeze–thaw cycles, the weight increase ratio of the specimen was the largest, at 0.19%, while after 100 freeze–thaw cycles, the weight increase ratio decreased to 0.03%. The main reason for the weight increase of A6M1PPF0.90 in the early stage of freeze–thaw cycles is as follows: the rise and fall in temperature inside the specimen during the freeze–thaw cycles caused the gas in the internal pores to be discharged, and the pores were filled with liquid [45]. At the same time, the surface of A6M1PPF0.90 caused by freeze–thaw cycles had very little peeling of the cementitious matrix.

As the number of freeze–thaw cycles continued to increase, the peeling of the cementitious matrix on the surface of the A6M1PPF0.90 increased, and the weight loss gradually increased. At 200 freeze–thaw cycles, the weight loss ratio was 1.98%, and at 225 freeze–thaw cycles, this value increased to 4.43%.

In summary, the addition of PPF significantly improved the frost resistance of AAFSC; from the perspective of weight loss, the freeze–thaw life of A6M1PPF0.90 was increased from 150 to 225 compared to A6M1, fully demonstrating that the addition of PPF significantly enhanced the frost resistance of AAFSC.

### 5.6. RDME of Concrete After Freeze–Thaw Cycles

**Figure 13:**
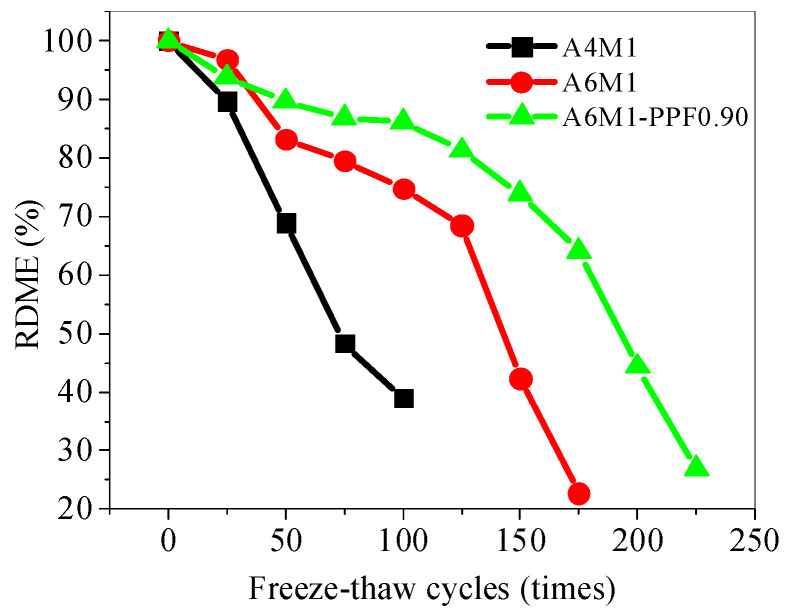
RDME ratio of AAFSC after freeze–thaw cycles.

Figure 13 shows the percentage of RDME of AAFSC during freeze–thaw cycles relative to the initial (i.e., 0 freeze–thaw cycles) RDME. It can be seen that A4M1, A6M1, and A6M1PPF0.90 have significant differences in the changes of RDME after different freeze–thaw cycles. The RDME of A4M1 rapidly decreased; after 50 freeze–thaw cycles, the RDME was 69% of the initial value, while after 100 freeze–thaw cycles, the value had decreased to 39%. The RDME of A6M1 decreased to 68.5% of its initial value after 125 freeze–thaw cycles, and the decrease in RDME after 150 freeze–thaw cycles was similar to that of A4M1 after 100 freeze–thaw cycles.

As the number of freeze–thaw cycles increased, the decrease in RDME of A6M1PPF0.90 slowed down significantly compared to A6M1. After 100 freeze–thaw cycles, more than 80% of the initial value remained, and after 175 freeze–thaw cycles, it was still higher than 60%.

### 5.7. Pore Characteristics of Concrete Deteriorated by Freeze–Thaw Cycles

Concrete is a complex heterogeneous multiphase composite material, and pores are a key component of its microstructure. These pores are mainly distributed in the hardened cementitious matrix and the interface transition zone between the cementitious matrix and the aggregate. The types of pores include gel pores and capillary pores, and a small amount of internal defects and microcracks exist in the cementitious matrix. These pores form a network inside the concrete, and their distribution changes due to changes in internal and external conditions.

Pores have a significant impact on the macroscopic properties of concrete, involving pore structure characteristics such as total porosity, pore size distribution, and pore morphology. Research suggests that the distribution characteristics of pores have a more significant impact on the macroscopic properties of concrete compared to porosity.

**Figure 14:**
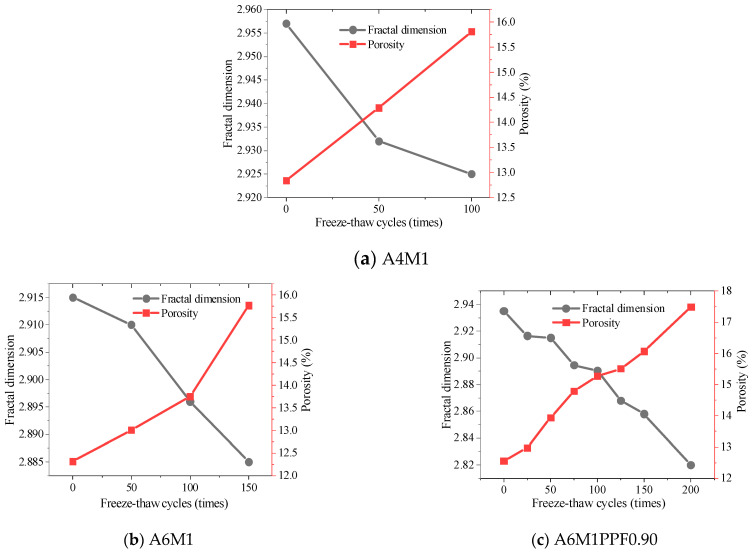
Relationship between freeze–thaw times of AAFSC and porosity and fractal dimension.

Figure 14 shows the changes in porosity and fractal dimension of the pore structure of A4M1, A6M1, and A6M1PPF0.90 after different freeze–thaw cycles. The porosity of A4M1, A6M1, and A6M1PPF0.90 increased with the increasing freeze–thaw cycles. The initial porosity of A4M1 at 0 freeze–thaw cycles was 12.84%, and after 100 freeze–thaw cycles, its porosity increased to 15.81%, representing an increase of 23.13% compared to 0 freeze–thaw cycles. The initial porosity of A6M1 was 12.31%, and the porosity after 100 freeze–thaw cycles was 13.75%, representing an increase of 11.7%. The initial porosity of A6M1PPF0.90 was 12.56%, and after 100 freeze–thaw cycles, the porosity was 15.27%. After 200 freeze–thaw cycles, the porosity of A6M1PPF0.90 was 17.49%, representing an increase of 39.25% compared to the value before freeze–thaw cycles.

After freeze–thaw cycles, the pore structure of the cementitious matrix of A4M1, A6M1, and A6M1PPF0.90 exhibited distinct fractal characteristics. The fractal dimension range of A4M1 was from 2.925 to 2.957, A6M1 ranged from 2.885 to 2.915, and A6M1PPF0.90 ranged from 2.82 to 2.935. As the number of freeze–thaw cycles increased, the fractal dimension of the concrete decreased, indicating a reduction in the complexity of the pore structure and an increase in the proportion of large pores.

The engineering properties of concrete materials, such as strength, permeability, and frost resistance, are closely related to their pore structure. A.M. Neville, the authoritative expert in the field of concrete, indicated that pore characteristics could be used to predict various properties of concrete [46]. M. Röβler et al. [47] summarized the relationship between the compressive strength and porosity of porous materials, as shown in Table 3.

Where $f_{c}$ is the compressive strength when porosity is P, $f_{c0}$ is the compressive strength when porosity is 0; $P_{0}$ is the porosity when the strength is 0; and $k_{b}$, $k_{r}$, $k_{s},$ and $k_{h}$ are experimental constants.

**Figure 15:**
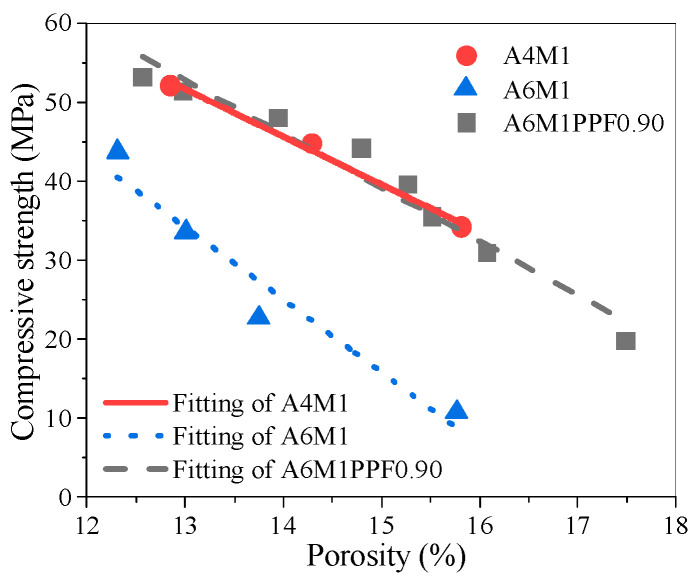
Compressive strength and porosity of AAFSC after freeze–thaw cycles.

The relationship between the porosity and compressive strength of A4M1, A6M1, and A6M1PPF0.90 after different freeze–thaw cycles is shown in Figure 15. By using the linear fitting method, the relationship model was determined as shown in Table 4. The model conformed to the Hasselmann equation. After verification, only the Hasselmann equation could accurately describe the relationship between the porosity and compressive strength of AAFSC under freeze–thaw degradation conditions among the equations listed in Table 3.

Where $f_{c}$ is the compressive strength, P is the porosity.

**Figure 16:**
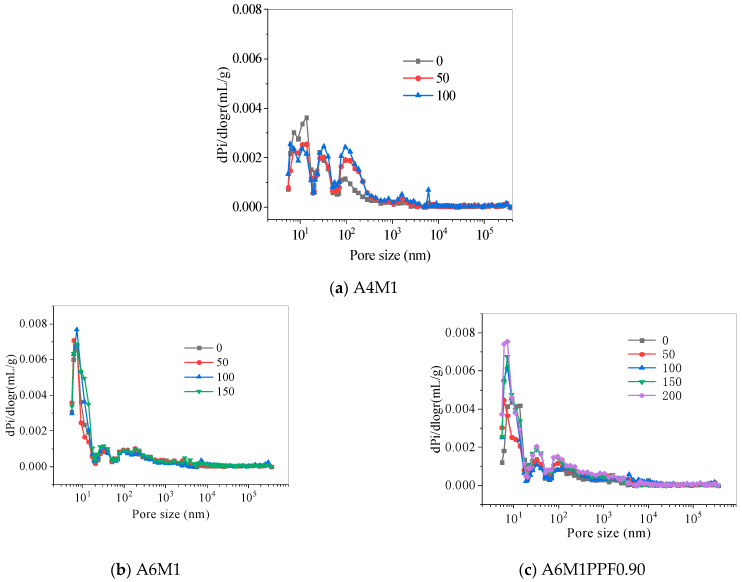
Distribution of pore size of AAFSC after freeze–thaw cycles.

The pore size distribution curve of AAFSC after freeze–thaw cycles is shown in Figure 16. The pore size distribution range of AAFSC was from 5.5 nm to 360,000 nm. Wu Zhongwei [48] classified the pores of concrete into four categories based on the different effects of pore size on concrete strength, as shown in Table 5.

According to Figure 16, as the number of freeze–thaw cycles increased from 0 to 100, the proportion of pores smaller than 20 nm in A4M1 decreased from 30.5% to 17.16%, the proportion of pores between 20 nm and 50 nm decreased from 22.87% to 17.71%, the proportion of pores between 50 nm and 200 nm increased from 21.26% to 31.21%, and the proportion of pores larger than 200 nm increased from 25.38% to 33.93%. As the number of freeze–thaw cycles increased from 0 to 150, the proportion of harmless pores in A6M1 showed no significant change, and the proportion of less harmful pores did not change significantly. The proportion of harmful pores decreased from 17.4% to 13.44%, while the proportion of multiple harmful pores increased from 36.5% to 40.37%. As the number of freeze–thaw cycles increased from 0 to 200, the proportion of pores smaller than 20 nm decreased from 36.17% to 31.83%, the proportion of pores between 20 nm and 50 nm decreased from 15.03% to 12.12%, the proportion of pores between 50 nm and 200 nm increased from 16.28% to 19.18%, and the proportion of pores larger than 200 nm increased from 32.52% to 36.86%.

Overall, as the number of freeze–thaw cycles increased, the proportion of harmless and less harmful pores in AAFSC decreased, while the proportion of harmful and multiple harmful pores increased, resulting in a loose overall pore structure of the cementitious matrix and a gradual deterioration of its various properties.

## 6. Conclusions

This research prepared high fly ash content AAFSC at room temperature, and systematically studied the improvement effect of adding PPF on its mechanical properties and freeze–thaw durability. The following main conclusions were obtained.

1. Based on the evaluation of compressive strength and flexural strength, the optimal dosage of PPF was 0.90%. The addition of PPF increased the 28-day flexural strength of concrete by 3.98%. The 1-year compressive strength of A6M1PPF0.90 reached 54.9 MPa, which was 161% of the strength at 28 days. Compared to A6M1, its compressive strength had increased by 2.81%.

2. The analysis of the frost resistance indicators such as surface morphology, residual compressive strength, weight loss, and RDME of AAFSC after different freeze–thaw cycles showed that when the alkali equivalent of the alkali activator was increased from 4% to 6%, the frost resistance of the concrete was improved. Furthermore, by adding 0.90% PPF, the frost resistance of the concrete after freeze–thaw cycles was further significantly improved. After 175 freeze–thaw cycles, the RDME of A6M1PPF0.90 was still higher than 60%.

3. With the increase in freeze–thaw cycles, the porosity of AAFSC increased. The pore structure of the alkali-activated cementitious matrix after freeze–thaw cycles exhibited distinct fractal characteristics, with fractal dimensions ranging from 2.82 to 2.957. The fractal dimension decreased with the increase in freeze–thaw cycles, indicating a decrease in the complexity of concrete pore structure and an increase in the proportion of large pores. The analysis of pore size distribution further indicated that the proportion of harmless and less harmful pores decreased, while the proportion of harmful and multiple harmful pores increased. The relationship model between the porosity and compressive strength of AAFSC after freeze–thaw cycles was established in the article.

Finally, limitations and considerations for future work will be discussed. The modulus of the activator used in this article was fixed at 1, and further research is needed to investigate the effect of alkali activator modulus on the performance of AAFSC. In addition, it is necessary to derive and establish a freeze–thaw damage model to conduct a more in-depth analysis of the freeze–thaw damage mechanism of AAFSC.

## Figures and Tables

**Table 1:** Chemical components of fly ash and slag [42].

Chemical Components (wt%)	Fly Ash	Slag
SiO_2_	58.23	29.51
Al_2_O_3_	19.21	15.32
Fe_2_O_3_	8.2	0.85
CaO	7.12	40.63
K_2_O	2.29	0.46
MgO	1.46	7.82
Na_2_O	1.32	0.41
TiO_2_	0.78	1.4
SO_3_	0.54	2.74
P_2_O_5_	0.25	-
MnO	-	0.53
Other Components	0.6	0.33

**Table 2:** Mix proportions of AAFSC.

MIX ID	Fly Ash (kg/m^3^)	Slag (kg/m^3^)	Fine Aggregate (kg/m^3^)	Coarse Aggregate (kg/m^3^)	Sodium Silicate (kg/m^3^)	Sodium Hydroxide (kg/m^3^)	Water (kg/m^3^)	Water Reducer (kg/m^3^)	PPF (%)
A4M1	344	86	655	1165	54.5	12.3	120.4	3.01	-
A6M1	344	86	655	1165	81.7	18.4	105.3	3.01	-
A8M1	344	86	655	1165	109	24.6	90.2	3.01	-
A6M1PPF0.45	344	86	655	1165	81.7	18.4	105.3	4.73	0.45
A6M1PPF0.90	344	86	655	1165	81.7	18.4	105.3	4.73	0.90
A6M1PPF1.35	344	86	655	1165	81.7	18.4	105.3	4.73	1.35

**Table 3:** Relationship between compressive strength and porosity of porous materials [47].

Name of Equation	Expression
Balshin equation	$f_{c}=f_{c0}{\left(1-P\right)}^{k_{b}}$
Ryshkevitch and Duckworth equation	$f_{c}=f_{c0}e^{{-k}_{r}P}$
Schiller equation	$f_{c}=k_{s}ln(P_{0}/P)$
Hasselmann equation	$f_{c}=f_{c0}-k_{h}P$

**Table 4:** Relationship between the porosity and compressive strength of AAFSC after freeze–thaw cycles.

MIX ID	Relationship Between Porosity and Compressive Strength	R^2^
A4M1	$f_{c}=130.13-6.03P$	0.9916
A6M1	$f_{c}=153.66-9.19P$	0.9403
A6M1PPF0.90	$f_{c}=141.11-6.79P$	0.9592

**Table 5:** Wu Zhongwei’s classification of concrete pore size [48].

Classification of Pores	Harmless Pores	Less Harmful Pores	Harmful Pores	Multiple Harmful Pores
Pore size	<20 nm	20–50 nm	50–200 nm	>200 nm

## Data Availability

Data is contained within the article, The original contributions presented in this study are included in the article. Further inquiries can be directed to the corresponding author.

## References

[1] Gartner E. (2004). Industrially interesting approaches to “low-CO_2_” cements. Cem. Concr. Res..

[2] Souayfan F., Rozière E., Loukili A., Justino C. (2023). Effect of Retarders on the Reactivity and Hardening Rate of Alkali-Activated Blast Furnace Slag Grouts. Materials.

[3] Amran YH M., Alyousef R., Alabduljabbar H., Justino C. (2020). Clean production and properties of geopolymer concrete; A review. J. Clean. Prod..

[4] Lehne J., Preston F. (2018). Making Concrete Change. Innovation in Low-Carbon Cement and Concrete.

[5] Rashad A.M. (2020). Effect of steel fibers on geopolymer properties—The best synopsis for civil engineer. Constr. Build. Mater..

[6] Provis J.L., Deventer J. (2014). Alkali Activated Materials.

[7] Wang S. (2023). Experimental Study on Self Shrinkage Characteristics and MgO Shrinkage Reduction Characteristics of Alkali Activated Slag Cementitious Materials. Master’s Thesis.

[8] Kumarappa D.B., Peethamparan S., Ngami M. (2018). Autogenous shrinkage of alkali activated slag mortars: Basic mechanisms and mitigation methods. Cem. Concr. Res..

[9] Ding Y., Dai J.-G., Shi C.-J. (2016). Mechanical properties of alkali-activated concrete: A state-of-the-art review. Constr. Build. Mater..

[10] Cartwright C., Rajabipour F., Radlińska A. (2014). Shrinkage Characteristics of Alkali-Activated Slag Cements. J. Mater. Civ. Eng..

[11] Li H., Zhuge L., Shi S., Xu D. (2012). The hydration products of fly ash based cementitious materials stimulated by NaOH. J. Ceram..

[12] Prinya C., Ubolluk R. (2023). Calcium wastes as an additive for a low calcium fly ash geopolymer. Sci. Rep..

[13] Wongpaun A., Tangchirapat W., Suwan T., Fan M. (2023). Factors affecting compressive strength and expansion due to alkali-silica reaction of fly ash-based alkaline activated mortar. Case Stud. Constr. Mater..

[14] Rafeet A., Vinai R., Soutsos M., Sha W. (2019). Effects of slag substitution on physical and mechanical properties of fly ash-based alkali activated binders (AABs). Cem. Concr. Res..

[15] Zhang D.-W., Sun X.-M., Li H. (2023). Relationship between macro-properties and amorphous gel of FA-based AAMs with different curing conditions after elevated temperature. Ceram. Int..

[16] Guo X., Shi H., Dick W.A. (2010). Compressive strength and microstructural characteristics of class C fly ash geopolymer. Cem. Concr. Compos..

[17] Zhu H., Zhang Y., Yu C., Qiao P., Li H. (2022). Mechanical properties of alkali activated fly ash cementitious materials during microwave curing stage. J. Build. Mater..

[18] Somaratna J., Ravikumar D., Neithalath N. (2010). Response of alkali activated fly ash mortars to microwave curing. Cem. Concr. Res..

[19] Chindaprasirt P., Rattanasak U., Taebuanhuad S. (2013). Role of microwave radiation in curing the fly ash geopolymer. Adv. Powder Technol..

[20] Graytee A., Sanjayan G.J., Nazari A. (2018). Development of a high strength fly ash-based geopolymer in short time by using microwave curing. Ceram. Int..

[21] Zhang Y. (2019). Experimental Study on the Influence of Microwave Heating on the Mechanical Properties of Alkali Excited High Calcium Fly Ash. Master’s Thesis.

[22] Shi S., Li H., Zhou Q., Zhang H., Basheer P.A.M., Bai Y. (2023). Alkali-activated fly ash cured with pulsed microwave and thermal oven: A comparison of reaction products, microstructure and compressive strength. Cem. Concr. Res..

[23] Jiao X. (2019). Preparation of Alkali Activated Fly Ash Based Concrete and Exploration of Its High Temperature Resistance Performance. Master’s Thesis.

[24] Ziolkowski M., Kovtun M. (2017). Confined-Direct Electric Curing of NaOH-activated fly ash based brick mixtures under free drainage conditions: Part 1. Factorial experimental design. Constr. Build. Mater..

[25] Ürünveren H., Beycïoğlu A., Resuloğulları Ç.E., Dïşken N.B. (2024). A comparative investigation of eco-friendly fly ash-based geopolymer mortar produced by using electrical and heat curing: Mechanical properties, energy consumption and cost. Constr. Build. Mater..

[26] Jiao X., Li H., Zheng W., Zhu H., Wy F., Wang W., Zhou M. (2019). Study on the Effect of DC Pre curing on the Early Strength of NaOH Induced Fly Ash Silicates Bulletin. Bull. Chin. Ceram. Soc..

[27] Nodehi M., Ozbakkaloglu T., Gholampour A., Mohammed T., Shi X. (2022). The effect of curing regimes on physico-mechanical, microstructural and durability properties of alkali-activated materials: A review. Constr. Build. Mater..

[28] Nath P., Sarker P.K. (2014). Effect of GGBFS on setting, workability and early strength properties of fly ash geopolymer concrete cured in ambient condition. Constr. Build. Mater..

[29] Jimena MH D., María C. (2022). Influence of the Fly Ash Content on the Fresh and Hardened Properties of Alkali-Activated Slag Pastes with Admixtures. Materials.

[30] Luo X., Xu J., Bai E., Li W. (2012). Systematic study on the basic characteristics of alkali-activated slag-fly ash cementitious material system. Constr. Build. Mater..

[31] Sun Y., Liu Z., Ghorbani S., Ye G., De Schutter G. (2022). Fresh and hardened properties of alkali-activated slag concrete: The effect of fly ash as a supplementary precursor. J. Clean. Prod..

[32] Srinivasamurthy L., Chevali V.S., Zhang Z., Wang H. (2021). Phase changes under efflorescence in alkali activated materials with mixed activators. Constr. Build. Mater..

[33] Aiken T.A., Kwasny J., Sha W., Tong K.T. (2021). Mechanical and durability properties of alkali-activated fly ash concrete with increasing slag content. Constr. Build. Mater..

[34] Lee N., Lee H. (2016). Influence of the slag content on the chloride and sulfuric acid resistances of alkali-activated fly ash/slag paste. Cem. Concr. Compos..

[35] Wang X., Kong L., Zhao W., Liu Y. (2023). Chloride transport resistance of alkali-activated concrete exposed to combined chloride, sulfate and carbonation environment. Constr. Build. Mater..

[36] Prabu B., Kumutha R., Vijai K. (2017). Effect of fibers on the mechanical properties of fly ash and GGBS based geopolymer concrete under different curing conditions. Indian J. Eng. Mater. Sci..

[37] Farhan N.A., Sheikh M.N., Hadi M.N.S. (2018). Engineering Properties of Ambient Cured Alkali-Activated Fly Ash–Slag Concrete Reinforced with Different Types of Steel Fiber. J. Mater. Civ. Eng..

[38] Kuranlı F., Uysal M., Abbas M.T., Çoşgun T., Niş A., Aygörmez Y., Canpolat O., Al-Mashhadani M.M. (2023). Mechanical and durability properties of steel, polypropylene and polyamide fiber reinforced slag-based alkali-activated concrete. Eur. J. Environ. Civ. Eng..

[39] Yuan Y., Zhao R., Li R., Wang Y., Cheng Z., Li F., Ma Z.J. (2024). Frost resistance of fiber-reinforced blended slag and Class F fly ash-based geopolymer concrete under the coupling effect of freeze-thaw cycling and axial compressive loading. Constr. Build. Mater..

[40] Amran M., Fediuk R., Abdelgader H.S., Murali G., Ozbakkaloglu T., Lee Y.H., Lee Y.Y. (2022). Fiber-reinforced alkali-activated concrete: A review. J. Build. Eng..

[41] Yuan Z., Jia Y. (2021). Mechanical properties and microstructure of glass fiber and polypropylene fiber reinforced concrete: An experimental study. Constr. Build. Mater..

[42] Yuan Z., Jia Y., Sun J., Zhang X., Hu Y., Han X. (2024). Study on the Properties of High Fly Ash Content Alkali-Activated Fly Ash Slag Pastes and Fiber-Reinforced Mortar Under Normal Temperature Curing. Materials.

[43] (2019). Standard for Test Method of Mechanical Properties on Ordinary Concrete.

[44] (2009). Standard for Test Methods of Long-Performance and Durability of Ordinary Concrete.

[45] Wang X.X., Liu C., Yin L.Q., Yan C.W., Liu S.G. (2021). Natural pumice concrete freeze-thaw damage and life prediction model. Silic. Bull..

[46] Neville A.M. (2012). Properties of Concrete.

[47] Röβler M., Odler I. (1985). Investigations on the relationship between porosity structure and strength of hydrated portland cement pastes III. Effect of clinker composition and gypsum addition. Cem. Concr. Res..

[48] Wu Z. (1999). High Performance Concrete.

